# The Effect of a Housing First Intervention on Acute Health Care Utilization among Homeless Adults with Mental Illness: Long-term Outcomes of the At Home/Chez-Soi Randomized Pragmatic Trial

**DOI:** 10.1007/s11524-021-00550-1

**Published:** 2021-06-28

**Authors:** James Lachaud, Cilia Mejia-Lancheros, Anna Durbin, Rosane Nisenbaum, Ri Wang, Patricia O’Campo, Vicky Stergiopoulos, Stephen W. Hwang

**Affiliations:** 1 MAP Centre for Urban Health Solutions, Li Ka Shing Knowledge Institute, St Michael’s Hospital, 209 Victoria St, Toronto, ON M5B 1T8 Canada; 2 Department of Psychiatry, University of Toronto, Toronto, ON Canada; 3 Applied Health Research Centre, Li Ka Shing Knowledge Institute, St Michael’s Hospital, Toronto, ON Canada; 4 Dalla Lana School of Public Health, University of Toronto, Toronto, ON Canada; 5 Centre for Addiction and Mental Health, Toronto, ON Canada; 6 Department of Medicine, University of Toronto, Toronto, ON Canada

**Keywords:** Homeless persons, Mental illness, Housing First, Hospitalization, Emergency department visit

## Abstract

We assessed the effects of the Toronto Site Housing First (HF) intervention on hospitalizations and emergency department (ED) visits among homeless adults with mental illness over 7 years of follow-up. The Toronto Site is part of an unblinded multi-site randomized pragmatic trial of HF for homeless adults with mental illness in Canada, which followed participants up to 7 years. Five hundred seventy-five participants were recruited and classified as having high (HN) or moderate need (MN) for mental health support services. Each group was randomized into intervention (HF) and treatment as usual groups, and 567 (98.6%) consented to link their data to health administrative databases. HF participants received a monthly rent supplement of $600 (Canadian) and assertive community treatment (ACT) support or intensive care management (ICM) support based on need level. Treatment as usual (TAU) participants had access to social, housing, and health services generally available in the community. Outcomes included all-cause and mental health-specific hospitalization, number of days in hospital, and ED visit. We used GEE models to estimate ratio of rate ratios (RRR). The results showed HF with ACT had no significant effect on hospitalization rates among HN participants, but reduced the number of days in hospital (RRR = 0.32, 95% CI 0.16-0.63) and number of ED visits (RRR = 0.57, 95% CI 0.34-0.95). HF with ICM resulted in an increase in the number of hospitalizations (RRR = 1.69, 95% CI 1.09-2.60) and ED visit rates (RRR = 1.42, 95% CI 1.01-2.01) but had no effect in days in hospital for MN participants. Addressing the health needs of this population and reducing acute care utilization remain system priorities. Trial registration: http://www.isrctn.com/identifier: ISRCTN42520374

**Supplementary Information:**

The online version contains supplementary material available at 10.1007/s11524-021-00550-1.

## Introduction

People experiencing homelessness are affected by chronic and acute medical conditions [1–8], neurocognitive impairment, and substance use disorders at higher rates than the general population [1, 9–11]. The unstable living conditions associated with homelessness not only increase heath need complexity but in addition interfere with accessing primary health care and effective disease management. It is therefore not surprising that, individuals experiencing homelessness are higher users of emergency departments (ED) [12] and are more frequently admitted to hospital compared to the general population [2, 12–14]. The provision of stable housing with supportive services has been identified as a promising intervention to decrease ED visits and hospital admissions for this population [15]. However, controlled studies of housing interventions have shown mixed effects on health care utilization [16–19]. These studies have generally used self-reported data on health care use, analyzed only a few specific health care services, or have had a relatively short period of follow-up. These limitations have resulted in uncertainty regarding the long-term impacts of supportive housing programs on health care utilization.

The purpose of this analysis of the Toronto Site At Home/Chez-Soi randomized pragmatic study is to investigate the effects of a Housing First (HF) intervention, including immediate access to housing and mental health support services, on several acute health care utilization outcomes, including all-cause hospitalization, mental health-related hospitalizations, and ED visits, among adults experiencing homelessness and mental illness over a 7-year follow-up period. This analysis leverages comprehensive provincial administrative databases and the longest HF trial to date to ascertain the health utilization outcomes of study participants over an extended follow-up period in a large urban setting with universal health insurance.

## Methods

### Design and Setting

The present study is a secondary exploratory analysis of the At Home/Chez-Soi (AH|CS) study, Toronto Site, which was part of a multi-site pragmatic randomized trial examining an HF intervention for homeless adults with mental illness in five cities across Canada: Vancouver, Winnipeg, Toronto, Montreal, and Moncton [20]. In contrast to traditional approaches that require homeless individuals to first accept treatment and placement in transitional housing before accessing permanent housing, HF interventions offer homeless individuals immediate access to permanent housing in conjunction with mental health support services. This paper reports findings from the Toronto Site of the At Home/Chez-Soi study. The Full AH|CS trial protocol and the Toronto AH|CS site primary results have been published elsewhere [20, 21].

### Participants

The study design, recruitment methods, instruments used, and inclusion criteria have been reported in previous publications [20, 22]. Briefly, participants were included if they fulfilled the following criteria: (1) were ≥18 years old; (2) experienced absolute homelessness or precarious housing; and (3) experienced a mental illness as determined using the Mini International Neuropsychiatric Interview 6.0 (MINI), with or without a co-existing substance use disorder [20, 21]. Individuals were excluded if they were not legally residing in Canada or were already receiving support services equivalent to those provided by the intervention.

Out of 1342 referred candidates, a total of 575 Toronto AH|CS’ participants met eligibility criteria and were recruited from October 1, 2009, to June 2011 and followed over a period of 24 months after randomization. Subsequently, the study received additional funding to extend the intervention and participant follow-up up for a further 4 years. Hence, participants were further followed from January 1, 2014, to March 31, 2017, for an overall long-term follow-up period of up to 7 years.

At baseline, participants were classified as having high need (HN) if they fulfilled all three of the following criteria: (1) having a current psychotic disorder or bipolar disorder based on the MINI; (2) having a Multnomah Community Ability Scale (MCAS) score of 62 or lower, which indicates at least moderate disability; and (3) at least one of three conditions: 2 or more hospitalizations for mental illness in any 1 of the last 5 years, recent arrest or incarceration, or co-morbid substance use based on the MINI. All other participants were classified as having moderate needs (MN) [20, 23].

### Intervention

Using a computerized adaptive randomization algorithm implemented by the central data collection system [20], HN participants were randomly assigned to either treatment as usual (TAU) or HF with assertive community treatment (ACT) support plus monthly rent supplement (C$ 600). ACT included an inter-professional team offering nursing and medical care, case management support, and peer support with 24/7 coverage [20]. Participants assigned to TAU had access to social housing and services that were generally available in the community. MN participants were randomly assigned to either treatment as usual (TAU) or the HF with intensive case management (ICM) support plus a monthly rent supplement of C$ 600. ICM services included case management and referral to other services as needed [24, 25].

**Fig. 1:**
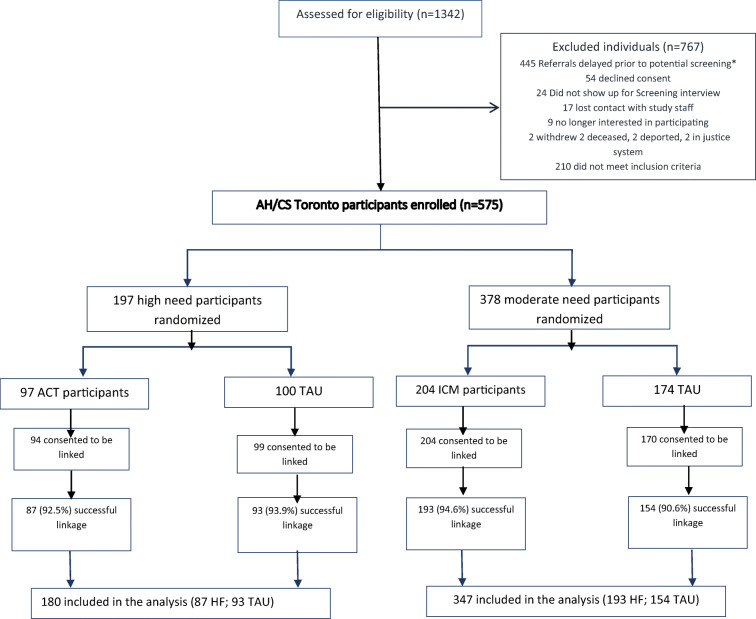
Study flowchart. *Referrals were kept for a period of up to 3 months. During the early phases of the study, there were more referrals than staff available to accept new participants; therefore, many referrals were delayed beyond the 3-month period and were excluded prior to screening

Assuming an attrition rate of 40%, a minimum sample size at baseline of at least 100 participants per intervention group was estimated to have an 80% power to detect a medium effect size (Cohen d = 0.5) [20]. The enrolled, allocated, and analyzed number of participants are shown in Fig. 1.

### Ethical Approvals

All study participants provided written consent to participate in the study. The study has been approved by the Research Ethics Board at St. Michael’s Hospital in Toronto, Canada. The study has been registered with the International Standard Randomized Control Trial Number Register (ISRCTN 42520374).

### Health Administrative Data

Study data were linked with health administrative data and analyses at ICES, which houses administrative health data over the entire Ontario population. Data for all-cause hospitalization were extracted from the Discharge Abstract Database (DAD). Mental health hospitalizations were derived from the Ontario Mental Health Reporting System (OMHRS) combined with mental health admissions in the DAD. Emergency department visits were retrieved from the National Ambulatory Care Reporting System (NACRS). These datasets were linked using unique encoded identifiers and analyzed at ICES.

### Outcome Measures

The main outcomes of this analysis were all-cause hospitalization incidence and number of hospitalizations, mental health hospitalization incidence and number of hospitalizations, number of days in hospital, and ED visit incidence and number of visits. The outcomes were extracted from administrative data over the following observation period: (1) the 1-year period prior to randomization; (2) 0-2-year post-randomization (i.e., the 24-month follow-up period planned in the original study design); (3) 2-7-year post-randomization (i.e., the extended follow-up period); and (4) 0-7-year post-randomization (i.e., the entire follow-up period).

### Analysis

We analyzed outcomes among all participants and stratified by level of need for mental health services (HN and MN). First, for each observation period, we computed (1) incidence rate (IR), using the first hospitalization or ED visit, and the time-at-risk (calculated in person-years) at the end of each observation period or censored for death or withdrawal from the study; and (2) rate of the total number of hospitalization, number of days hospitalized, and ED visits over each observation period, and the time-at-risk at the end of the analyzed observation periods, or censored at the date of death or withdrawal from the study.

Second, to assess the impact of the intervention on health care utilization rates, we considered both treatment group and time period. We used generalized estimating equation (PROCGENDMOD) models and chose the health care utilization outcome distribution by comparing their mean and variance. We included HF intervention group, time period, and the interaction term between treatment group and time to determine whether the program had a differential impact on the outcome change. For all the estimated models, analyses were conducted at the individual level, and the unstructured covariance matrix was used in all models. An offset equal to the natural log of person-years was included in all models to account for differential time-at-risk for each participant at each observation period. Based on the outcome dispersion, a Poisson distribution was used. Ratios of rate ratios (RRR), comparing prior-randomization and post-randomization rate ratios and 95% CIs and a p-value, were estimated. SAS version 9.4 was used for all analyses (SAS Institute Inc. 2013. SAS® 9.4 Statements: Reference. Cary, NC: SAS Institute Inc.).

## Results

### Characteristics of Participants

Out of 575 study participants, 567 (98.6%) provided consent to link their survey data with administrative ICES data, and 527 (92.9%) were successfully linked for these analyses. Of these, 180 (34.2%) were from the HN group (HF, n = 87 vs. TAU, n = 93) and 347 (65.8%) from the MN group (HF, n = 193 vs. TAU, n = 154) (Fig. 1). Comparison between our final sample and the not linked participants showed no significant differences regarding sociodemographic characteristics (Supplementary Information Table A1, available on-line).

Tables 1 and 2 present the characteristics of the 527 participants with linked administrative data, by level of need and intervention group at baseline. All sociodemographic characteristics and mental health diagnoses were similar between treatment groups, except for the HN group, where there was a lower mean age and a higher proportion of females in the HF group. Health care utilization patterns over 1 year prior the baseline are depicted by randomized groups and level of need in the Supplementary Information (see Table A2 to A5, available on-line).

**Table 1:** Baseline Socioeconomic characteristics of study participants with linked administrative data, by level of need and treatment group

	High Needs (*N*=180)				Moderate Needs (*N*=347)			
		HF (*n*=87)	TAU (*n*=93)			HF (*n*=193)	TAU (*n*=154)	
	n	% or mean ± SD	% or mean ± SD	*p*-value	n	% or mean ± SD	% or mean ± SD	*p*-value
Demographics								
Age (years)	180	37.67±11.06	41.45±12.03	0.030	347	39.56±11.60	40.79±12.51	0.345
Gender, self-reported								
Male	129	57 (65.5%)	72 (77.4%)	0.042	234	131 (67.9%)	103 (66.9%)	0.931
Female^a^	49	30 (34.5%)	21 (22.6%)		113	62 (32.1%)	51 (33.1%)	
Self-Identified ethno-racial group (self-reported)								
Others	43	18 (20.7%)	25 (26.9%)	0.451	120	66 (34.2%)	54 (35.1%)	0.068
Black	55	30 (34.5%)	25 (26.9%)		115	73 (37.8%)	42 (27.3%)	
White	82	39 (44.8%)	43 (46.2%)		112	54 (28.0%)	58 (37.7%)	
Socioeconomic factors								
Marital status								
Single	127	65 (74.7%)	62 (66.7%)	0.428	231	128 (66.3%)	103 (66.9%)	0.883
Other	43	19 (21.8%)	24 (25.9%)		112	63 (32.6%)	49 (31.8%)	
Education								
Less than high school	82	41 (47.1%)	41 (44.1%)	0.566	164	98 (50.8%)	66 (42.9%)	0.328
Completed high school	33	15 (17.2%)	18 (19.4%)		63	31 (16.1%)	32 (20.8%)	
Some post-secondary school	51	29 (33.3%)	22 (23.7%)		112	61 (31.6%)	51 (33.1%)	
Homelessness during lifetime (years)								
<3 years	65	35 (40.2%)	30 (32.3%)	0.543	171	97 (50.3%)	74 (48.1%)	0.663
≥3years	104	51 (58.6%)	53 (57.0%)		171	93 (48.2%)	78 (50.6%)	

*a*= Includes seven transsexual or transgender participants

**Table 2.** Mental health disorders of study participants with linked administrative data, by level of need and treatment group at baseline

	High Needs (*N*=180)				Moderate Needs (*N*=347)			
		HF (*n*=87)	TAU (*n*=93)			HF (*n*=87)	TAU (*n*=93)	
	n	% or mean ± SD	% or mean ± SD	*p*-value	n	% or mean ± SD	% or mean ± SD	*p*-value
Mental health and substance use^a^								
Major depressive episode								
No	148	72 (82.8%)	76 (81.7%)	0.856	192	106 (54.9%)	86 (55.8%)	0.864
Yes	32	15 (17.2%)	17 (18.3%)		155	87 (45.1%)	68 (44.2%)	
Manic or hypomanic episode								
No	163	76 (87.4%)	87 (93.5%)	0.156	309	171 (88.6%)	138 (89.6%)	0.765
Yes	17	11 (12.6%)	6 (6.5%)		38	22 (11.4%)	16 (10.4%)	
Post-traumatic stress disorder								
No	157	74 (85.1%)	83 (89.2%)	0.400	250	137 (71.0%)	113 (73.4%)	0.622
Yes	23	13 (14.9%)	10 (10.8%)		97	56 (29.0%)	41 (26.6%)	
Panic disorder								
No	172	82 (94.3%)	90 (96.8%)	0.412	281	157 (81.3%)	124 (80.5%)	0.845
Yes	8	1 to 5 (0.6 to 5.8%)^b^	1 to 5 (1.1% to 5.5%)^b^		66	36 (18.7%)	30 (19.5%)	
Mood disorder with psychotic features								
No	134	65 (74.7%)	69 (74.2%)	0.936	282	158 (81.9%)	124 (80.5%)	0.750
Yes	46	22 (25.3%)	24 (25.8%)		65	35 (18.1%)	30 (19.5%)	
Psychotic disorder								
No	74	37 (42.5%)	37 (39.8%)	0.709	260	145 (75.1%)	115 (74.7%)	0.923
Yes	106	50 (57.5%)	56 (60.2%)		87	48 (24.9%)	39 (25.3%)	
Substance use disorder								
No	161	75 (86.2%)	86 (92.5%)	0.172	318	175 (90.7%)	143 (92.9%)	0.465
Yes	19	12 (13.8%)	7 (7.5%)		29	18 (9.3%)	11 (7.1%)	
Alcohol abuse								
No	148	70 (80.5%)	78 (83.9%)	0.550	304	165 (85.5%)	139 (90.3%)	0.181
Yes	32	17 (19.5%)	15 (16.1%)		43	28 (14.5%)	15 (9.7%)	

*a*= Based on the DSM-IV criteria using the Mini International Neuropsychiatric Interview [MINI] version 6.0, *c*=Absolute numbers less than six have been suppressed to reduce the risk of identification

Over the 7-year follow-up period, participants had similar incidence rate for all-cause hospitalization (HF: 0.23, 95% CI [0.20-0.27] vs. TAU: 0.20, 95% CI [0.17-0.23] per person year) or for ED visits (HF: 0.63, 95% CI [0.55-0.71] vs. TAU: 0.64, 95% CI [0.56-0.73]). However, compared to the TAU group, HF participants had lower number of days in hospital (HF: 7.82, 95% CI [7.69-7.95] vs. TAU: 10.74, 95% CI [10.58-10.91]) and lower number of ED visits (HF: 3.28, 95% CI [3.19-3.36] vs. TAU: 4.07 95% CI [3.97-4.17]). When analyzing the incidence and count rates by level of needs for mental health services, HN participants had higher acute health care utilization rates compared to MN participants, regardless of their intervention group (HF or TAU) (see Table A2 to A2 in the Supplementary Information, available on-line).

Table 3 shows the intervention group by time interaction results from models comparing each post-randomization period (0-2 years, 2-7 years, 0-7 years) with 1-year pre-randomization. Over the 7 years of follow-up, the HF intervention had differential effects on hospitalizations and ED visits according to the need level of participants. In the HN group, HF was not associated with changes in all-cause or mental health incidence hospitalization rates. However, HF was associated with reduction in the number of days in hospital (ratio of rate ratio—RRR = 0.32 95% CI: 0.16-0.63) and the number of ED visits (RRR = 0.57 95% CI: 0.34-0.95) compared to TAU.

**Table 3:** Treatment and time interaction results from models comparing each post-randomization period with 1 year pre-randomization

Outcome	Randomization groups	0 to 2 years post-randomization vs	2 to 7 years post-randomization vs	0 to 7 years post-randomization vs
		1 year pre-randomization	1 year pre-randomization	1 year pre-randomization
		Ratio of rate ratios (95%CI)	Ratio of rate ratios (95%CI)	Ratio of rate ratios (95%CI)
**Hospitalization**	All Interventions vs. All Treatment as Usual	0.98 (0.72-1.33)	1.32 (0.95-1.84)	1.15 (0.85-1.56)
	HN-HF vs. TAU	0.70 (0.45-1.07)	0.89 (0.52-1.52)	0.83 (0.52-1.35)
	MN-HF vs. TAU	1.31 (0.82-2.10)	**1.66 (1.06-2.61)**	1.40 (0.92-2.13)
**Number of hospitalizations**	All Interventions vs. All Treatment as Usual	1.10 (0.79-1.52)	1.20 (0.81-1.76)	1.16 (0.83-1.62)
	HN-HF vs. TAU	0.69 (0.45-1.04)	0.87 (0.49-1.55)	0.80 (0.49-1.30)
	MN-HF vs. TAU	**1.80 (1.13-2.88)**	**1.64 (1.01-2.70)**	**1.69 (1.09-2.60)**
**Mental health Hospitalization**	All Interventions vs. All Treatment as Usual	1.06 (0.75-1.50)	1.28 (0.88-1.87)	1.15 (0.83-1.60)
	HN-HF vs. TAU	0.72 (0.46-1.15)	0.82 (0.47-1.45)	0.71 (0.44-1.16)
	MN-HF vs. TAU	1.61 (0.91-2.86)	**1.87 (1.07-3.27)**	**1.66 (1.01-2.74)**
**Number of mental health Hospitalizations**	All Interventions vs. All Treatment as Usual	1.08 (0.72-1.63)	1.13 (0.71-1.80)	1.11 (0.74-1.65)
	HN-HF vs. TAU	0.62 (0.37-1.04)	0.77 (0.41-1.45)	0.71 (0.42-1.22)
	MN-HF vs. TAU	**1.99 (1.11-3.56)**	1.79 (0.95-3.38)	**1.86 (1.09-3.17)**
**Number of days in hospital**	All Interventions vs. All Treatment as Usual	0.62 (0.38-1.03)	**0.42 (0.22-0.77)**	**0.48 (0.29-0.81)**
	HN-HF vs. TAU	**0.45 (0.24-0.83)**	**0.28 (0.12-0.66)**	**0.32 (0.16-0.63)**
	MN-HF vs. TAU	1.81 (0.96-3.39)	0.99 (0.43-2.29)	1.29 (0.63-2.65)
**Emergency Department (ED) visit**	All Interventions vs. All Treatment as Usual	1.02 (0.78-1.34)	1.26 (0.95-1.65)	1.10 (0.82-1.48)
	HN-HF vs. TAU	0.63 (0.39-1.03)	0.75 (0.44-1.27)	0.57 (0.31-1.03)
	MN-HF vs. TAU	1.29 (0.92-1.80)	**1.59 (1.15-2.20)**	**1.42 (1.01-2.01)**
**Number of ED visits**	All Interventions vs. All Treatment as Usual	0.89 (0.65-1.22)	0.82 (0.52-1.29)	0.84 (0.60-1.19)
	HN-HF vs. TAU	0.75 (0.44-1.26)	**0.49 (0.25-0.94)**	**0.57 (0.34-0.95)**
	MN-HF vs. TAU	1.12 (0.81-1.53)	**1.51 (1.02-2.23)**	1.36 (0.99-1.88)

**Bold** means “statistically significant at a level of 5%”

On the other hand, for the MN group, HF was associated with an increase in all-cause of incidence hospitalizations (RRR = 1.69 (95% CI: 1.09-2.60)), mental health hospitalizations (RRR = 1.66 (95% CI: 1.01-2.74)), and ED visit rates (RRR = 1.42 (95% CI: 1.01-2.01)). HF was not associated with the number of days in hospital.

Similar results were found where 0-2 years and 2-7 years to 1-year pre-randomization were compared.

## Discussions

This study, leveraging 7-year follow-up data from a randomized trial of Housing First, examined the long-term effects of HF interventions on acute health care utilization among homeless adults with mental illness by linking to administrative provincial health records. Findings revealed that the effects of the intervention differed substantially by participant baseline need levels and service support intensity. For participants with high needs at baseline, receiving HF with ACT support, there were no significant effects on all-cause and mental health hospitalizations, but a significant reduction in the number of days in hospital as well as the number of ED visits. A study of an HF intervention in four French cities among high-need participants similarly found no significant effects on hospitalization or ED visits, but reduced inpatient days [26]. Another randomized study of the effects of permanent supportive housing for chronically homeless high users of multiple systems in Santa Clara County, California (USA) found no effects on ED or inpatient care use [27].

For individuals with moderate needs at baseline, receiving HF with ICM, the intervention resulted in an increase in the number of all-cause and mental health hospitalizations, and ED visits, but had no statistically significant effect on days in hospital, compared to TAU participants. Previous analyses similarly highlighted mixed effects of housing interventions on health outcomes and health care utilization [28, 29] when analyzing high and moderate-need participants separately. These analyses found a reduction in ED visits among HN participants, compared to the TAU group, and no effect among MN participants over a 2-year period [30, 31]. Differential effects between high and moderate groups were also found for other outcomes, such as quality of life scores [32], food security [33], or rapid and stable rehousing [29, 34].

Several factors inherent to the HF intervention could underline these findings. Prior AH|CS analyses demonstrated that HF can facilitate rapid rehousing among the MN group, contrarily to the HN group [29, 34]. Rapid rehousing enables MN participants to shift priorities, from housing and survival to other basic needs, including health care for chronic comorbidities that may require both preventive and acute care. This may have contributed to the increase of the number of all-cause hospitalizations (RRR = 1.80 95% CI: 1.13-2.88) and mental health hospitalization (RRR = 1.99 95% CI: 1.11-3.56) during the 0-2-year post-randomization.

The ICM support provided to MN participants, based on need level at enrolment, seems limited to cope with these new challenges, compared to ACT services for HN participants. Contrary to ICM, the ACT model included intensive support available 7 days/week and 24 h/day, including psychiatric support [25, 35]. A recent systematic review of the effects of different case management intervention models on health and social outcomes of homeless populations highlighted that intensive case management (ICM), offered to moderate-need participants, had limited and mixed effects on participants’ hospitalization outcomes [25]. ICM interventions resulted in small reductions in the number of ED visits in some settings, but showed no effect on the utilization of other hospital services, when compared to usual care [36–38]. However, the effects of ACT interventions on hospitalization outcomes were mainly positive, reducing by half the number days in hospital, compared to standard case management [39] or resulting in fewer ED visits and number of days in hospital compared to usual care [25, 40].

The strengths of our study included a rigorous randomized controlled design, long duration of follow-up, use of administrative databases to ascertain hospitalization and ED visits with an extremely high level of completeness, and the ability to compare effects in participants with both moderate and high support needs.

Nonetheless, our study has certain limitations. First, the study focused specifically on homeless adults with mental illness and with access to Ontario Health Insurance Plan, and our findings may not be applicable to other homeless populations. A second limitation is that the support services provided to high-need and moderate-need groups were different, and it is not possible to determine if the different outcomes observed in these groups were due to the type of services provided, the baseline characteristics of the participants, or a combination of the two. Finally, regarding the generalisability of our results, the study was conducted in a country with universal health care and a broad social safety network. Thus, TAU participants were able to access to housing, social and economic services in the community, potentially reducing the apparent effectiveness of the intervention.

## Conclusion

In summary, the provision of immediate housing and ICM support to homeless adults with moderate need for mental health services was associated with increased hospitalization rates and ED visits, but had no effect on the days in hospital. In contrast, a Housing First intervention providing immediate housing and ACT support had no effect on hospitalization rates among homeless adults with high need for mental health services, but reduced the number of days in hospital and ED visits. These findings demonstrate the importance of addressing the health and support needs of this population, especially those that may not qualify for ACT services that may require more comprehensive and coordinated supports, than those available through ICM.

## Supplementary Information

ESM 1 (DOCX 59 kb)

## Declarations

### Data Access, Responsibility, and Analysis

Drs Hwang and Lachaud had full access to all of the data in the study and take responsibility for the integrity of the data and the accuracy of the data analysis.

### Data Sharing Statement

The dataset from this study is held securely in coded form at ICES. While legal data sharing agreements between ICES and data providers (e.g., healthcare organizations and government) prohibit ICES from making the dataset publicly available, access may be granted to those who meet pre-specified criteria for confidential access, available at www.ices.on.ca/DAS (email: das@ices.on.ca). The full dataset creation plan and underlying analytic code are available from the authors upon request, understanding that the computer programs may rely upon coding templates or macros that are unique to ICES and are therefore either inaccessible or may require modification.

## References

[1] Fazel S, Geddes JR, Kushel M (2014). The health of homeless people in high-income countries: descriptive epidemiology, health consequences, and clinical and policy recommendations. Lancet..

[2] Hwang SW, Chambers C, Chiu S, Katic M, Kiss A, Redelmeier DA, Levinson W (2013). A comprehensive assessment of health care utilization among homeless adults under a system of universal health insurance. Am J Public Health.

[3] Mackelprang JL, Graves JM, Rivara FP (2014). Homeless in America: injuries treated in US emergency departments, 2007-2011. Int J Inj Control Saf Promot.

[4] Baggett TP, O’Connell JJ, Singer DE, Rigotti NA (2010). The unmet health care needs of homeless adults: a national study. Am J Public Health.

[5] Ferenchick GS (1990). The medical problems of homeless clinic patients: a comparative study. J Gen Intern Med.

[6] Gelberg L, Linn LS (1989). Assessing the physical health of homeless adults. JAMA J Am Med Assoc.

[7] Savage CL, Lindsell CJ, Gillespie GL, Dempsey A, Lee RJ, Corbin A (2006). Health care needs of homeless adults at a nurse-managed clinic. J Community Health Nurs.

[8] Beijer U, Wolf A, Fazel S (2012). Prevalence of tuberculosis, hepatitis C virus, and HIV in homeless people: a systematic review and meta-analysis. Lancet Infect Dis.

[9] Hwang SW (2001). Homelessness and health. Cmaj..

[10] Fazel S, Khosla V, Doll H, Geddes J (2008). The prevalence of mental disorders among the homeless in Western countries: systematic review and meta-regression analysis. PLoS Med.

[11] Hwang SW (2000). Mortality among men using homeless shelters in Toronto, Ontario. J Am Med Assoc.

[12] Chambers C, Chiu S, Katic M, et al. High utilizers of emergency health services in a population-based cohort of homeless adults. *Am J Public Health*. 2013;103(SUPPL. 2) 10.2105/AJPH.2013.301397. 10.2105/AJPH.2013.301397 PMC3969147 24148033

[13] Hwang S, Henderson M. Health care utilization in homeless people: translating research into policy and practice. *Agency Healthc Res Qual Work*. 2010;(10002):1–73. http://scholar.google.com/scholar?hl=en&btnG=Search&q=intitle:Health+Care+Utilization+in+Homeless+People:+Translating+Research+into+Policy+and+Practice#1. Accessed Jan 2021

[14] Clark RE, Weinreb L, Flahive JM, Seifert RW (2018). Health care utilization and expenditures of homeless family members before and after emergency housing. Am J Public Health.

[15] National Academies of Sciences, Engineering and M. Permanent supportive housing: evaluating the evidence for improving health outcomes among people experiencing chronic homelessness. The National Academies Press.; 2018. doi:10.17226/25133 30148580

[16] Mackelprang JL, Collins SE, Clifasefi SL (2014). Housing first is associated with reduced use of emergency medical services. Prehospital Emerg Care.

[17] Larimer ME, Malone DK, Garner MD, Atkins DC, Burlingham B, Lonczak HS, Tanzer K, Ginzler J, Clifasefi SL, Hobson WG, Marlatt GA (2009). Health care and public service use and costs before and after provision of housing for chronically homeless persons with severe alcohol problems. JAMA - J Am Med Assoc.

[18] Wright BJ, Vartanian KB, Li H-F, Royal N, Matson JK (2016). Formerly homeless people had lower overall health care expenditures after moving into supportive housing. Health Aff.

[19] Fenwick E, Macdonald C, Thomson H (2013). Economic analysis of the health impacts of housing improvement studies: a systematic review. J Epidemiol Community Health.

[20] Goering PN, Streiner DL, Adair C, Aubry T, Barker J, Distasio J, Hwang SW, Komaroff J, Latimer E, Somers J, Zabkiewicz DM (2011). The at Home/Chez Soi trial protocol: a pragmatic, multi-site, randomised controlled trial of a Housing First intervention for homeless individuals with mental illness in five Canadian cities. BMJ Open.

[21] Hwang SW, Stergiopoulos V, O’Campo P, Gozdzik A (2012). Ending homelessness among people with mental illness: the at Home/Chez Soi randomized trial of a Housing First intervention in Toronto. BMC Public Health.

[22] Canadian Population Health Initiative of the Canadian Institute for Health. Mental health, mental illness, and homelessness in Canada (200(. In: Hulchanski JD, Campsie P, Chau SBY, Hwang SW, Paradis E, eds. Finding home: policy options for addressing homelessness in Canada (*e‐book*). Toronto (Canada), Cities Centre, University of Toronto; 2009:306-353. https://www.homelesshub.ca/FindingHome. Accessed Jan 2021.

[23] Stergiopoulos V, Hwang SW, Gozdzik A, Nisenbaum R, Latimer E, Rabouin D, Adair CE, Bourque J, Connelly J, Frankish J, Katz LY, Mason K, Misir V, O’Brien K, Sareen J, Schütz CG, Singer A, Streiner DL, Vasiliadis HM, Goering PN (2015). Effect of scattered-site housing using rent supplements and intensive case management on housing stability among homeless adults with mental illness: a randomized trial. JAMA - J Am Med Assoc.

[24] De Vet R, Van Luijtelaar MJA, Brilleslijper-Kater SN, Vanderplasschen W, Beijersbergen MD, Wolf JRLM. Effectiveness of case management for homeless persons: a systematic review. *Am J Public Health*. 2013;103(10) 10.2105/AJPH.2013.301491. 10.2105/AJPH.2013.301491 PMC3780754 23947309

[25] Ponka D, Agbata E, Kendall C, et al. The effectiveness of case management interventions for the homeless, vulnerably housed and persons with lived experience: a systematic review. *PLoS One*. 2020;15(4) 10.1371/journal.pone.0230896. 10.1371/journal.pone.0230896 PMC7313544 32271769

[26] Tinland A, Loubière S, Boucekine M, Boyer L, Fond G, Girard V, Auquier P (2020). Effectiveness of a housing support team intervention with a recovery-oriented approach on hospital and emergency department use by homeless people with severe mental illness: a randomised controlled trial. Epidemiol Psychiatr Sci.

[27] Raven MC, Niedzwiecki MJ, Kushel M (2020). A randomized trial of permanent supportive housing for chronically homeless persons with high use of publicly funded services. Health Serv Res.

[28] Hwang SW, Tolomiczenko G, Kouyoumdjian FG, Garner RE (2005). Interventions to improve the health of the homeless: a systematic review. Am J Prev Med.

[29] Stergiopoulos V, Mejia-Lancheros C, Nisenbaum R, Wang R, Lachaud J, O'Campo P, Hwang SW (2019). Long-term effects of rent supplements and mental health support services on housing and health outcomes of homeless adults with mental illness: extension study of the At Home/Chez Soi randomised controlled trial. Lancet Psychiatry.

[30] Goldfinger SM, Schutt RK, Tolomiczenko GS (1999). Housing placement and subsequent days homeless among formerly homeless adults with mental illness. Psychiatr Serv..

[31] Aubry T, Nelson G, Tsemberis S (2015). Housing first for people with severe mental illness who are homeless: a review of the research and findings from the At Home-Chez soi demonstration project. Can J Psychiatr Rev Can Psychiatr.

[32] Aubry T, Goering P, Veldhuizen S, Adair CE, Bourque J, Distasio J, Latimer E, Stergiopoulos V, Somers J, Streiner DL, Tsemberis S (2016). A multiple-city RCT of housing first with assertive community treatment for homeless Canadians with serious mental illness. Psychiatr Serv.

[33] O’Campo P, Hwang SW, Gozdzik A, Schuler A, Kaufman-Shriqui V, Poremski D, Lazgare LIP, Distasio J, Belbraouet S, Addorisio S (2017). Food security among individuals experiencing homelessness and mental illness in the At Home/Chez Soi Trial. Public Health Nutr.

[34] Lachaud J, Mejia-Lancheros C, Nisenbaum R, Stergiopoulos V, O’Campo P, Hwang SW. Housing First model and severe mental disorders: the challenge of exiting homelessness. Ann Am Acad Pol Soc Sci. Published online 2020:In Press.

[35] Bond GR, Drake RE, Mueser KT, Latimer E (2001). Assertive community treatment for people with severe mental illness: critical ingredients and impact on patients. Dis Manag Heal Outcomes.

[36] Rosenblum A, Nuttbrock L, McQuistion H, Magura S, Joseph H (2002). Medical outreach to homeless substance users in New York City: preliminary results. Subst Use Misuse.

[37] Shumway M, Boccellari A, O’Brien K, Okin RL (2008). Cost-effectiveness of clinical case management for ED frequent users: results of a randomized trial. Am J Emerg Med.

[38] Malte CA, Cox K, Saxon AJ (2017). Providing intensive addiction/housing case management to homeless veterans enrolled in addictions treatment: a randomized controlled trial. Psychol Addict Behav.

[39] Essock SM, Frisman LK, Kontos NJ (1998). Cost-effectiveness of assertive community treatment teams. Am J Orthop.

[40] Essock SM, Mueser KT, Drake RE, Covell NH, McHugo GJ, Frisman LK, Kontos NJ, Jackson CT, Townsend F, Swain K (2006). Comparison of ACT and standard case management for delivering integrated treatment for co-occurring disorders. Psychiatr Serv.

