# Supplementary material for: The Effect of a Housing First Intervention on Acute Health Care Utilization among Homeless Adults with Mental Illness: Long-term Outcomes of the At Home/Chez-Soi Randomized Pragmatic Trial
Source: J Urban Health. 2021 Jun 28;98(4):505–15. doi: 10.1007/s11524-021-00550-1 (PMC8382791; doi:10.1007/s11524-021-00550-1)
Supplement: Supplementary file 1 — (DOCX 59 kb) [file 11524_2021_550_MOESM1_ESM.docx]

**Appendix**

**Table A1: Comparison of the main study population characteristics between those linked and those who did not link to ICES records.**

|  | **Participants linked to ICES records (n=527)** | | **Participants were not linked to ICES record (n=40)** | | |
| --- | --- | --- | --- | --- | --- |
|  | **n** | **%** | **n** | **%** | **p-value*** |
| **Intervention group** |  |  |  |  |  |
| HF | 280 | 53.1% | 18 | 45.0% | 0.321 |
| TAU | 247 | 46.9% | 22 | 55.0% |  |
| **Need group** |  |  |  |  |  |
| High Needs | 180 | 34.2% | 13 | 32.5% | 0.831 |
| Moderate Needs | 347 | 65.8% | 27 | 67.5% |  |
| **Intervention group by level of need** |  |  |  |  |  |
| **High Needs** |  |  |  |  | 0.418 |
| HN-HF-ACT | 87 | 16.5% | 7 | 17.5% |  |
| HN-TAU | 93 | 17.6% | 6 | 15.0% |  |
| **Demographics** |  |  |  |  |  |
| Age (years) | 39.94 ± 11.89 | | 38.23 ± 11.02 | | 0.378 |
| **Gender, self-reported** |  |  |  |  |  |
| Male | 363 | 68.9% | 25 | 62.5% | 0.775 |
| Female | 164 | 31.1% | 15 | 37.5% |  |
| **Self-Identified ethno-racial group (self-reported)** | | |  |  |  |
| Others | 163 | 30.9% | 15 | 37.5% | 0.098 |
| Black | 170 | 32.3% | 17 | 42.5% |  |
| White | 194 | 36.8% | 8 | 20.0% |  |
| **Country of birth** |  |  |  |  |  |
| Other | 227 | 43.1% | 20 | 50.0% | 0.111 |
| Canada | 280 | 53.1% | 14 | 35.0% |  |
| Missing | 20 | 3.8% | 6 | 15.0% |  |
| **Marital status** |  |  |  |  |  |
| Single/never married | 358 | 67.9% | 25 | 62.5% | 0.838 |
| Other^a^ | 155 | 29.4% | 5-10 | 12.5 to 25.0% |  |
| Missing^a^ | 14 | 2.7% | 1-5 | 2.5 to 12.5% |  |
| **Education** |  |  |  |  |  |
| Less than high school | 246 | 46.7% | 14 | 35.0% | 0.430 |
| Completed high school | 96 | 18.2% | 6 | 15.0% |  |
| Some post-secondary school^a^ | 163 | 30.9% | 10-15 | 25.0 to 37.5% |  |
| Missing^a^ | 22 | 4.2% | 1-5 | 2.5 to 12.5% |  |
| **Homelessness during lifetime (years)** |  |  |  |  |  |
| <3 years | 236 | 44.8% | 19 | 47.5% | 0.272 |
| ≥3years | 275 | 52.2% | 15 | 37.5% |  |
| Missing | 16 | 3.0% | 6 | 15.0% |  |
| **Mental health disorders (MINI)** |  |  |  |  |  |
| Major depressive episode |  |  |  |  |  |
| No | 340 | 64.5% | 24 | 60.0% | 0.566 |
| Yes | 187 | 35.5% | 16 | 40.0% |  |
| Manic or hypomanic episode |  |  |  |  |  |
| No^a^ | 472 | 89.6% | 30-35 | 75.0 to 87.5% | 0.683 |
| Yes^a^ | 55 | 10.4% | 1-5 | 2.5 to 12.5% |  |
| Post-traumatic stress disorder |  |  |  |  |  |
| No | 407 | 77.2% | 28 | 70.0% | 0.297 |
| Yes | 120 | 22.8% | 12 | 30.0% |  |
| Panic disorder |  |  |  |  |  |
| No^a^ | 453 | 86.0% | 30-35 | 75.0 to 87.5% | 0.786 |
| Yes^a^ | 74 | 14.0% | 1-5 | 2.5 to 12.5% |  |
| Mood disorder with psychotic features |  |  |  |  |  |
| No | 416 | 78.9% | 32 | 80.0% | 0.874 |
| Yes | 111 | 21.1% | 8 | 20.0% |  |
| Psychotic disorder |  |  |  |  |  |
| No | 334 | 63.4% | 21 | 52.5% | 0.170 |
| Yes | 193 | 36.6% | 19 | 47.5% |  |
| Substance use disorder |  |  |  |  |  |
| No^a^ | 479 | 90.9% | 31-36 | 77.5 to 90.0% | 0.851 |
| Yes^a^ | 48 | 9.1% | 1-5 | 2.5 to 12.5% |  |
| Alcohol abuse |  |  |  |  |  |
| No^a^ | 452 | 85.8% | 31-36 | 77.5 to 90.0% | 0.456 |
| Yes^a^ | 75 | 14.2% | 1-5 | 2.5 to 12.5% |  |

a=Absolute numbers less than six have been suppressed to reduce the risk of identification.

**Table A2: Number of Hospitalizations and ED visits by randomization group and period of intervention**

| Variable Name |  | Randomization (Comparison) Groups | | | | | |
| --- | --- | --- | --- | --- | --- | --- | --- |
|  |  | Overall Comparison | | High Needs | | All Moderate Needs | |
|  |  | (all need levels) | |  |  |  |  |
|  | Overall sample | All HF | All TAU | HN-HF-ACT | HN-TAU | MN-HF-ICM | MN-TAU |
| **1-year pre-randomization period at the Toronto site** |  |  |  |  |  |  |  |
| **All-cause hospitalizations** |  |  |  |  |  |  |  |
| First hsp | 220 | 118 | 102 | 55 | 51 | 63 | 51 |
| Number of hsp | 448 | 250 | 198 | 120 | 111 | 130 | 87 |
| **Mental Hospitalizations** |  |  |  |  |  |  |  |
| First mhsp | 175 | 91 | 84 | 47 | 45 | 44 | 39 |
| Number of mhsp | 350 | 188 | 162 | 98 | 98 | 90 | 64 |
| **Emergency Department Visits** |  |  |  |  |  |  |  |
| First ED visit | 391 | 203 | 188 | 76 | 76 | 127 | 112 |
| Number of ED visit | 2692 | 1396 | 1296 | 749 | 686 | 647 | 610 |
| **0-2 year post-randomization period at the Toronto site** |  |  |  |  |  |  |  |
| **Hospitalizations** |  |  |  |  |  |  |  |
| First hsp | 229 | 123 | 106 | 55 | 62 | 68 | 44 |
| Number of hsp | 596 | 347 | 249 | 124 | 167 | 223 | 82 |
| **Mental Hospitalizations** |  |  |  |  |  |  |  |
| First mhsp | 155 | 83 | 72 | 39 | 47 | 44 | 25 |
| Number of mhsp | 367 | 205 | 162 | 72 | 115 | 133 | 47 |
| **Emergency Department Visits** |  |  |  |  |  |  |  |
| First ED visit | 403 | 212 | 191 | 72 | 85 | 140 | 106 |
| Number of ED visit | 4024 | 1974 | 2050 | 1032 | 1263 | 942 | 787 |
| **2-7 year post-randomization period at the Toronto site** |  |  |  |  |  |  |  |
| **Hospitalizations** |  |  |  |  |  |  |  |
| First hsp | 283 | 165 | 118 | 63 | 59 | 102 | 59 |
| Number of hsp | 1114 | 684 | 430 | 274 | 270 | 410 | 160 |
| **Mental Hospitalizations** |  |  |  |  |  |  |  |
| First mhsp | 174 | 100 | 74 | 41 | 44 | 59 | 30 |
| Number of mhsp | 626 | 363 | 263 | 152 | 183 | 211 | 80 |
| **Emergency Department Visits** |  |  |  |  |  |  |  |
| First ED visit | 422 | 230 | 192 | 75 | 83 | 155 | 109 |
| Number of ED visit | 7895 | 3817 | 4078 | 1577 | 2741 | 2240 | 1337 |
| **0-7 year post-randomization period at the Toronto site** |  |  |  |  |  |  |  |
| **Hospitalizations** |  |  |  |  |  |  |  |
| First hsp | 358 | 200 | 158 | 76 | 75 | 124 | 83 |
| Number of hsp | 1702 | 1027 | 675 | 396 | 433 | 631 | 242 |
| **Mental Hospitalizations** |  |  |  |  |  |  |  |
| First mhsp | 235 | 130 | 105 | 52 | 60 | 78 | 45 |
| Number of mhsp | 993 | 568 | 425 | 224 | 298 | 344 | 127 |
| **Emergency Department Visits** |  |  |  |  |  |  |  |
| First ED visit | 477 | 255 | 222 | 81 | 90 | 174 | 132 |
| Number of ED visit | 11919 | 5791 | 6128 | 2609 | 4004 | 3182 | 2124 |

**Table A3. person-years by randomization group and randomization period**

| Variable Name |  | Randomization (Comparison) Groups | | | | | |
| --- | --- | --- | --- | --- | --- | --- | --- |
|  |  | Overall Comparison | | High Needs | | All Moderate Needs | |
|  |  | (all need levels) | |  |  |  |  |
|  | Overall sample | All HF | All TAU | HN-HF-ACT | HN-TAU | MN-HF-ICM | MN-TAU |
| **1 year pre-randomization period at the Toronto site** |  |  |  |  |  |  |  |
| Time at Risk* | 527 | 280 | 247 | 87 | 93 | 193 | 154 |
| All-cause hospitalizations | 406.68 | 215.29 | 191.39 | 54.08 | 65.55 | 161.21 | 125.84 |
| Mental Hospitalizations | 431.52 | 230.14 | 201.37 | 60.56 | 67.88 | 169.59 | 133.49 |
| Emergency Department Visits | 278.77 | 153.22 | 125.55 | 34.13 | 39.45 | 119.09 | 86.10 |
| **0-2 year post-randomization period at the Toronto site** |  |  |  |  |  |  |  |
| Time at Risk* | 1046.41 | 557.91 | 488.50 | 174.33 | 185.81 | 383.58 | 302.69 |
| Hospitalizations | 743.31 | 397.82 | 345.49 | 96.86 | 99.46 | 300.96 | 246.03 |
| Mental Hospitalizations | 774.34 | 415.93 | 358.41 | 108.08 | 110.25 | 307.85 | 248.17 |
| Emergency Department Visits | 851.95 | 455.39 | 396.56 | 124.01 | 126.73 | 331.38 | 269.83 |
| Time at Risk* | 445.73 | 245.83 | 199.91 | 56.07 | 48.51 | 189.76 | 151.39 |
| **2-7 year post-randomization period at the Toronto site** |  |  |  |  |  |  |  |
| Time at Risk* | 2227.27 | 1210.26 | 1017.01 | 385.14 | 385.57 | 825.12 | 631.44 |
| All-cause hospitalizations | 1394.30 | 706.45 | 687.85 | 191.27 | 209.56 | 515.18 | 478.29 |
| Mental Hospitalizations | 1717.92 | 902.77 | 815.15 | 254.39 | 265.10 | 648.39 | 550.05 |
| Emergency Department Visits | 758.92 | 393.06 | 365.86 | 106.40 | 102.10 | 286.66 | 263.76 |
| **0-7 year post-randomization period at the Toronto site** |  |  |  |  |  |  |  |
| Time at Risk* | 3273.68 | 1768.17 | 1505.51 | 559.47 | 571.38 | 1208.70 | 934.14 |
| All-cause hospitalizations | 1660.95 | 855.45 | 805.50 | 177.10 | 191.67 | 678.35 | 613.84 |
| Mental Hospitalizations | 2221.19 | 1178.65 | 1042.54 | 297.79 | 289.39 | 880.86 | 753.16 |
| Emergency Department Visits | 750.75 | 405.44 | 345.31 | 92.15 | 67.41 | 313.29 | 277.90 |

person-years censoring for at first event occurrence (hospitalization or ED visit) or due to death or withdrawal if occurred before the end of the year

*person-years of observation with censoring occurring due to death, withdrawal from the study, or the end of the observation period

**Table A4.** **Incident and Total number event rates by randomization groups and randomization period**

| Variable Name | 1 year pre-randomization | | 0-2 years | | 2-7 years | | 0-7 years | |
| --- | --- | --- | --- | --- | --- | --- | --- | --- |
|  | All HF | All TAU | All HF | All TAU | All HF | All TAU | All HF | All TAU |
| **Hospitalizations** |  |  |  |  |  |  |  |  |
| Percent | 118 (42.1%) | 102 (41.3%) | 123 (43.9%) | 106 (42.9%) | 165 (58.9%) | 118 (47.8%) | 200 (71.4%) | 158 (64.0%) |
| Percent (95%CI) | 36.29 - 48.16 | 35.09 - 47.71 | 38.03 - 49.96 | 36.66 - 49.34 | 52.92 - 64.75 | 41.40 - 54.20 | 65.75 - 76.65 | 57.64 - 69.96 |
| IR | 0.55 | 0.53 | 0.31 | 0.31 | 0.23 | 0.17 | 0.23 | 0.2 |
| IR (95%CI) | 0.45 - 0.66 | 0.43 - 0.65 | 0.26 - 0.37 | 0.25 - 0.37 | 0.20 - 0.27 | 0.14 - 0.21 | 0.20 - 0.27 | 0.17 - 0.23 |
| Total Number rate | 0.89 | 0.8 | 0.62 | 0.51 | 0.57 | 0.42 | 0.58 | 0.45 |
| Total number rate (95%CI) | 0.79 - 1.01 | 0.69 - 0.92 | 0.56 - 0.69 | 0.45 - 0.58 | 0.52 - 0.61 | 0.38 - 0.46 | 0.55 - 0.62 | 0.42 - 0.48 |
| **Mental Hospitalizations** |  |  |  |  |  |  |  |  |
| Percent | 91 (32.5%) | 84 (34.0%) | 83 (29.6%) | 72 (29.2%) | 100 (35.7%) | 74 (30.0%) | 130 (46.4%) | 105 (42.5%) |
| Percent (95%CI) | 27.05 - 38.33 | 28.12 - 40.28 | 24.36 - 35.37 | 23.56 - 35.25 | 30.10 - 41.63 | 24.32 - 36.09 | 40.47 - 52.46 | 36.27 - 48.94 |
| IR | 0.4 | 0.42 | 0.18 | 0.18 | 0.11 | 0.09 | 0.11 | 0.1 |
| IR (95%CI) | 0.32 - 0.49 | 0.33 - 0.52 | 0.15 - 0.23 | 0.14 - 0.23 | 0.09 - 0.13 | 0.07 - 0.11 | 0.09 - 0.13 | 0.08 - 0.12 |
| Total Number rate | 0.67 | 0.66 | 0.37 | 0.33 | 0.3 | 0.26 | 0.32 | 0.28 |
| Total number rate (95%CI) | 0.58 - 0.77 | 0.56 - 0.77 | 0.32 - 0.42 | 0.28 - 0.39 | 0.27 - 0.33 | 0.23 - 0.29 | 0.30 - 0.35 | 0.26 - 0.31 |
| **Number of hospitalized days** |  |  |  |  |  |  |  |  |
| Total number rate | 14.9 | 10.98 | 9.32 | 10.8 | 7.13 | 10.71 | 7.82 | 10.74 |
| Total number rate los (95%CI) | 14.45 - 15.36 | 10.57 - 11.41 | 9.07 - 9.58 | 10.51 - 11.09 | 6.98 - 7.28 | 10.51 - 10.91 | 7.69 - 7.95 | 10.58 - 10.91 |
| **Emergency Room Visits** |  |  |  |  |  |  |  |  |
| Percent | 203 (72.5%) | 188 (76.1%) | 212 (75.7%) | 191 (77.3%) | 230 (82.1%) | 192 (77.7%) | 255 (91.1%) | 222 (89.9%) |
| Percent (95%CI) | 66.87 - 77.65 | 70.30 - 81.29 | 70.26 - 80.62 | 71.59 - 82.40 | 77.14 - 86.45 | 72.02 - 82.76 | 87.10 - 94.14 | 85.42 - 93.34 |
| IR | 1.32 | 1.5 | 0.86 | 0.96 | 0.59 | 0.52 | 0.63 | 0.64 |
| IR (95%CI) | 1.15 - 1.52 | 1.29 - 1.73 | 0.75 - 0.99 | 0.82 - 1.10 | 0.51 - 0.67 | 0.45 - 0.60 | 0.55 - 0.71 | 0.56 - 0.73 |
| Total Number rate | 4.99 | 5.25 | 3.54 | 4.2 | 3.15 | 4.01 | 3.28 | 4.07 |
| Total number rate (95%CI) | 4.73 - 5.25 | 4.97 - 5.54 | 3.38 - 3.70 | 4.02 - 4.38 | 3.05 - 3.26 | 3.89 - 4.13 | 3.19 - 3.36 | 3.97 - 4.17 |

**Table A5.** **Incident and Total number event rates by randomization groups, need level, and randomization period**

| **High need** |  |  |  |  |  |  |  |  |
| --- | --- | --- | --- | --- | --- | --- | --- | --- |
| Variable Name | 1 year pre | | 0-2 years | | 2-7 years | | 0-7 years | |
|  | All HF | All TAU | All HF | All TAU | All HF | All TAU | All HF | All TAU |
| **High need** |  |  |  |  |  |  |  |  |
| **Hospitalizations** |  |  |  |  |  |  |  |  |
| Percent | 55 (63.2%) | 51 (54.8%) | 55 (63.2%) | 62 (66.7%) | 63 (72.4%) | 59 (63.4%) | 76 (87.4%) | 75 (80.7%) |
| Percent (95%CI) | 52.20 - 73.31 | 44.17 - 65.19 | 52.20 - 73.31 | 56.13 - 76.11 | 61.79 - 81.46 | 52.81 - 73.19 | 78.50 - 93.52 | 71.15 - 88.11 |
| IR | 1.02 | 0.78 | 0.57 | 0.62 | 0.33 | 0.28 | 0.43 | 0.39 |
| IR (95%CI) | 0.77 - 1.32 | 0.58 - 1.02 | 0.43 - 0.74 | 0.48 - 0.80 | 0.25 - 0.42 | 0.21 - 0.36 | 0.34 - 0.54 | 0.31 - 0.49 |
| Total Number rate | 1.38 | 1.19 | 0.71 | 0.9 | 0.71 | 0.7 | 0.71 | 0.76 |
| Total number rate (95%CI) | 1.14 - 1.65 | 0.98 - 1.44 | 0.59 - 0.85 | 0.77 - 1.05 | 0.63 - 0.80 | 0.62 - 0.79 | 0.64 - 0.78 | 0.69 - 0.83 |
| **Mental Hospitalizations** |  |  |  |  |  |  |  |  |
| Percent | 47 (54.0%) | 45 (48.4%) | 39 (44.8%) | 47 (50.5%) | 41 (47.1%) | 44 (47.3%) | 52 (59.8%) | 60 (64.5%) |
| Percent (95%CI) | 43.00 - 64.77 | 37.89 - 58.99 | 34.15 - 55.87 | 39.97 - 61.07 | 36.33 - 58.13 | 36.86 - 57.94 | 48.71 - 70.15 | 53.91 - 74.17 |
| IR | 0.78 | 0.66 | 0.31 | 0.37 | 0.16 | 0.17 | 0.17 | 0.21 |
| IR (95%CI) | 0.57 - 1.03 | 0.48 - 0.89 | 0.22 - 0.43 | 0.27 - 0.49 | 0.12 - 0.22 | 0.12 - 0.22 | 0.13 - 0.23 | 0.16 - 0.27 |
| Total Number rate | 1.13 | 1.05 | 0.41 | 0.62 | 0.39 | 0.47 | 0.4 | 0.52 |
| Total number rate (95%CI) | 0.91 - 1.37 | 0.86 - 1.28 | 0.32 - 0.52 | 0.51 - 0.74 | 0.33 - 0.46 | 0.41 - 0.55 | 0.35 - 0.46 | 0.46 - 0.58 |
| **Number of hospitalized days** |  |  |  |  |  |  |  |  |
| Total number rate | 32.09 | 19 | 15.22 | 24.46 | 10.4 | 22.32 | 11.9 | 23.03 |
| Total number rate los (95%CI) | 30.91 - 33.30 | 18.12 - 19.91 | 14.64 - 15.81 | 23.75 - 25.18 | 10.08 - 10.72 | 21.85 - 22.80 | 11.62 - 12.19 | 22.63 - 23.42 |
| **Emergency Room Visits** |  |  |  |  |  |  |  |  |
| Percent | 76 (87.4%) | 76 (81.7%) | 72 (82.8%) | 85 (91.4%) | 75 (86.2%) | 83 (89.3%) | 81 (93.1%) | 90 (96.8%) |
| Percent (95%CI) | 78.50 - 93.52 | 72.35 - 88.98 | 73.16 - 90.02 | 83.75 - 96.21 | 77.15 - 92.66 | 81.11 - 94.72 | 85.59 - 97.43 | 90.86 - 99.33 |
| IR | 2.23 | 1.93 | 1.28 | 1.75 | 0.7 | 0.81 | 0.88 | 1.34 |
| IR (95%CI) | 1.75 - 2.79 | 1.52 - 2.41 | 1.00 - 1.62 | 1.40 - 2.17 | 0.55 - 0.88 | 0.65 - 1.01 | 0.70 - 1.09 | 1.07 - 1.64 |
| Total Number rate | 8.61 | 7.38 | 5.92 | 6.8 | 4.09 | 7.11 | 4.66 | 7.01 |
| Total number rate (95%CI) | 8.00 - 9.25 | 6.83 - 7.95 | 5.56 - 6.29 | 6.43 - 7.18 | 3.89 - 4.30 | 6.85 - 7.38 | 4.49 - 4.85 | 6.79 - 7.23 |
| **Moderate need** |  |  |  |  |  |  |  |  |
| **Hospitalizations** |  |  |  |  |  |  |  |  |
| Percent | 63 (32.6%) | 51 (33.1%) | 68 (35.2%) | 44 (28.6%) | 102 (52.9%) | 59 (38.3%) | 124 (64.3%) | 83 (53.9%) |
| Percent (95%CI) | 26.08 - 39.75 | 25.75 - 41.15 | 28.51 - 42.42 | 21.59 - 36.40 | 45.55 - 60.06 | 30.60 - 46.48 | 57.05 - 71.00 | 45.69 - 61.95 |
| IR | 0.39 | 0.41 | 0.23 | 0.18 | 0.2 | 0.12 | 0.18 | 0.14 |
| IR (95%CI) | 0.30 - 0.50 | 0.30 - 0.53 | 0.18 - 0.29 | 0.13 - 0.24 | 0.16 - 0.24 | 0.09 - 0.16 | 0.15 - 0.22 | 0.11 - 0.17 |
| Total Number rate | 0.67 | 0.56 | 0.58 | 0.27 | 0.5 | 0.25 | 0.52 | 0.26 |
| Total number rate (95%CI) | 0.56 - 0.80 | 0.45 - 0.70 | 0.51 - 0.66 | 0.22 - 0.34 | 0.45 - 0.55 | 0.22 - 0.30 | 0.48 - 0.56 | 0.23 - 0.29 |
| **Mental Hospitalizations** |  |  |  |  |  |  |  |  |
| Percent | 44 (22.8%) | 39 (25.3%) | 44 (22.8%) | 25 (16.2%) | 59 (30.6%) | 30 (19.5%) | 78 (40.4%) | 45 (29.2%) |
| Percent (95%CI) | 17.08 - 29.37 | 18.67 - 32.95 | 17.08 - 29.37 | 10.79 - 23.02 | 24.16 - 37.59 | 13.55 - 26.63 | 33.43 - 47.70 | 22.18 - 37.08 |
| IR | 0.26 | 0.29 | 0.13 | 0.09 | 0.09 | 0.05 | 0.09 | 0.06 |
| IR (95%CI) | 0.19 - 0.35 | 0.21 - 0.40 | 0.10 - 0.18 | 0.06 - 0.14 | 0.07 - 0.12 | 0.04 - 0.08 | 0.07 - 0.11 | 0.04 - 0.08 |
| Total Number rate | 0.47 | 0.42 | 0.35 | 0.16 | 0.26 | 0.13 | 0.28 | 0.14 |
| Total number rate (95%CI) | 0.37 - 0.57 | 0.32 - 0.53 | 0.29 - 0.41 | 0.11 - 0.21 | 0.22 - 0.29 | 0.10 - 0.16 | 0.26 - 0.32 | 0.11 - 0.16 |
| **Number of hospitalized days** |  |  |  |  |  |  |  |  |
| Total number rate | 7.16 | 6.14 | 6.64 | 2.41 | 5.6 | 3.62 | 5.93 | 3.23 |
| Total number rate los (95%CI) | 6.78 - 7.54 | 5.76 - 6.55 | 6.39 - 6.91 | 2.24 - 2.59 | 5.44 - 5.76 | 3.47 - 3.77 | 5.79 - 6.07 | 3.12 - 3.35 |
| **Emergency Room Visits** |  |  |  |  |  |  |  |  |
| Percent | 127 (65.8%) | 112 (72.7%) | 140 (72.5%) | 106 (68.8%) | 155 (80.3%) | 109 (70.8%) | 174 (90.2%) | 132 (85.7%) |
| Percent (95%CI) | 58.65 - 72.46 | 64.97 - 79.58 | 65.67 - 78.70 | 60.88 - 76.04 | 73.99 - 85.67 | 62.92 - 77.82 | 85.05 - 93.97 | 79.17 - 90.83 |
| IR | 1.07 | 1.3 | 0.74 | 0.7 | 0.54 | 0.41 | 0.56 | 0.47 |
| IR (95%CI) | 0.89 - 1.27 | 1.07 - 1.57 | 0.62 - 0.87 | 0.57 - 0.85 | 0.46 - 0.63 | 0.34 - 0.50 | 0.48 - 0.64 | 0.40 - 0.56 |
| Total Number rate | 3.35 | 3.96 | 2.46 | 2.6 | 2.71 | 2.12 | 2.63 | 2.27 |
| Total number rate (95%CI) | 3.10 - 3.62 | 3.65 - 4.29 | 2.30 - 2.62 | 2.42 - 2.79 | 2.60 - 2.83 | 2.01 - 2.23 | 2.54 - 2.73 | 2.18 - 2.37 |
